# Improving the quality of primary care by allocating performance-based targets, in a diverse insured population

**DOI:** 10.1186/s12913-016-1920-6

**Published:** 2016-11-21

**Authors:** Ronit Peled, Avi Porath, Rachel Wilf-Miron

**Affiliations:** 1 Department Health Systems Management, Faculty Health Sciences, Ben Gurion University of the Negev, Beer Sheva, 65321 Israel; 2 Peres Academy Center, Rehovot, Israel; 3 Department of Epidemiology, Faculty of Health Sciences, Ben Gurion University of the Negev, Beer Sheva, Israel; 4 The Gertner Institute for Epidemiology and Health Policy Research, Tel Aviv, Israel; 5 The School of Public Health, Sackler Faculty of Medicine, Tel Aviv University, Tel Aviv, Israel

**Keywords:** Public health, Quality improvement, Quality measurement, Quality indicators

## Abstract

**Background:**

Primary Care Health organizations, operating under universal coverage and a regulated package of benefits, compete mainly over quality of care. Monitoring, primary care clinical performance, has been repeatedly proven effective in improving the quality of care. In 2004, Maccabi Healthcare Services (MHS), the second largest Israeli HMO, launched its Performance Measurement System (PMS) based on clinical quality indicators. A unique module was built in the PMS to adjust for case mix while tailoring targets to the local units.

This article presents the concept and formulas developed to adjust targets to the units’ current performance, and analyze change in clinical indicators over a six year period, between sub-population groups.

**Methods:**

Six process and intermediate outcome indicators, representing screening for breast and colorectal cancer and care for patients with diabetes and cardiovascular disease, were selected and analyzed for change over time (2003–2009) in overall performance, as well as the difference between the lowest and the highest socio-economic ranks (SERs) and Arab and non-Arab members.

**Results:**

MHS demonstrated a significant improvement in the selected indicators over the years. Performance of members from low SERs and Arabs improved to a greater extent, as compared to members from high ranks and non-Arabs, respectively.

**Conclusion:**

The performance measurement system, with its module for tailoring of units' targets, served as a managerial vehicle for bridging existing gaps by allocating more resources to lower performing units. This concept was proven effective in improving performance while reducing disparities between diverse population groups.

## Background

Healthcare organizations operating under universal coverage, regulated prices and package of benefits compete mainly over quality of care and patient satisfaction [1, 2].

Monitoring clinical performance measures by large healthcare organizations has been repeatedly proven effective in improving the quality of care [3–6]. However, measurement alone is insufficient for improving quality. The Veteran Affairs (VA), for example, developed and maintained valid and cost-effective measures, but still demonstrates considerable variability in levels of performance across measures and facilities, suggesting the need for effective feedback to providers [7].

Achieving equity in health care is an important goal of most primary health care systems and reforms. Although primary health care plays an important role in improving health equity [8], international evidence shows that enhancement of primary primary healthcare services for vulnerable individuals (e.g. low income, diverse racial and ethnic populations) critical but not enough for reducing health and health care inequities [9–11]. Measuring and monitoring equity in healthcare settings remain challenging mainly because current primary healthcare indicators and measures do not adequately reflect organizations’ efforts to provide primary care services to groups most affected by structural inequities [12].

The VA has questioned the adequacy of their monitoring system using a "treat to target" method, which focuses on attainment of specific risk factor thresholds, in achieving control, in a diverse population. This concern has led the VA to develop a system that is based on “tightly linked” clinical action measures, in which the process specified by the measure is strongly tied to the evidence. Specifically, clinical action measures focus not only on the risk factor level but also give credit to the process [13]. In other healthcare systems, where performance is measured, a concern has been raised, that the results may account for differences in patient characteristics [14–16].

Research usually distinguishes between equality and equity in the provision of care and health outcomes: health equity is defined as the absence of systematic and potentially remediable differences in one or more characteristics of health across populations or population groups which are socially, economically, demographically or geographically defined [17]; whereas equality in health care means equal access, treatment and treatment outcomes for people of equal need [18]. These distinctions are important in developing equity-oriented indicators, given the widening inequities in health and social status.

The Israeli population is highly diverse. Twenty percent are Israeli Arabs who live mainly in rural settlements and mixed cities, and like all Israeli citizens, they are fully insured by the National Health Insurance Law under a universal care system. Eighteen percent of the Israeli population are considered "poor" with a 0.363 Gini Index for inequality in household income for 2013 [19]. Therefore, all Israeli health maintenance organizations (HMO) are challenged by this diversion, and are forced to invest in reducing inequalities and inequities.

This article has two objectives: 1) to describe the concept and implementation of a unique module, which adjusts performance targets for current achievements, in order to compensate for different case mixes and population diversion; and 2) to present an analysis of the effect of this concept on care quality and equity.

### Setting

This study was initiated by Maccabi Healthcare Services (MHS), the second largest Israeli health plan (HMO), that provides primary and secondary community-based services to two million beneficiaries. These services are provided to diverse population groups country-wide, through five regions, divided into 150 branches (the basic administrative unit). Services are based on a core staff of 8,000 physicians, including 2,000 primary care physicians, 1,200 nurses and other health professionals. Physicians are normally self-employed who work in MHS' clinics or in their private clinics with seventeen million encounters (in 2013). In-patient care is purchased by the MHS from local medical centers.

Nine percent of MHS members are categorized in the lowest quartile of socioeconomic status, while 25.6% are in the upper quartile. Seven percent of MHS members are Israeli Arabs who are entitle for a full healthcare coverage.

### Performance Measurement System (PMS)

In 2004 MHS launched a Performance Measurement System (PMS), aiming to improve quality of care by monitoring and internally reporting on clinical performance. Twenty-five process and intermediate outcome indicators represented six clinical domains: early detection of cancer (colon and breast); influenza and pneumococcal vaccination; early detection of risk factors and treatment for cardio-vascular diseases; diabetes care; diagnosis and treatment of depression; and appropriate use of antibiotics.

Each indicator definition was based on the National Program for Quality Indicators in Community Healthcare's definitions [20], which in turn was based on the Healthcare Effectiveness Data and Information Set (HEDIS) indicator set [3]. Indicators’ definitions are presented in Table 1. A steering committee consisted on: physicians; IT experts; central and regional quality management representatives evaluated the definitions which were finally approved by the organizational chief executive officer (CEO) and chief medical officer (CMO).

**Table 1:** Selected quality indicators and target populations^*^

Type of Care	Criteria for Meeting Quality Standards	Target Population in 2009 (N)
Mammography	Subjects: Females aged 52–74 with no history of cancer. Criterion: A mammography performed within the last 24 months.	153,785
Colorectal cancer screening	Subjects: Adults aged 51–74 with no history of cancer. Criterion: an FOBT^††^ within the last 12 months or a colonoscopy within the last 5 years	308,929
HbA1C Performance	Subjects: Adults recorded in the diabetes registry Criterion: HbA1C performed at least once during the last 12 months	77,898
Diabetes adequate control	Subjects: Adults recorded in the diabetes registry Criterion: HbA1C < 7 gr%, last test	77,898
Diabetes poor control	Subjects: Adults recorded in the diabetes registry Criterion: HbA1C > 9 gr%, last test.	77,898
Adequate control of LDL cholesterol, patients with CVD	Subjects: Adults recorded in the cardio-vascular disease registry Criterion: LDL < 100 mg%, last test	54,095

^*^Indicators were defined by MHS performance system in accordance with the National Quality Indicator Program's definitions

The core of the PMS is a unique module, built into the system, which allows setting tailor-made benchmarks for each of MHS' administrative units (regions and branches). The system produces monthly reports, which are distributed in a transparent manner to local managers. The reports reflect each unit's gaps between achieved and desired targets.

### Programming

MHS is a fully computerized healthcare organization: all diagnoses, prescriptions, diagnostic tests and billings are stored in a central data warehouse, from which the PMS retrieves information. A Microsoft Performance Point Server acts as a mediating platform supporting the integration of data, reports and explanatory documents.

### Setting units' tailor-made targets

The main objective of setting tailor-made targets, was to continually support and encourage the successful units but also to help and push, for better achievements, the weaker units.

For each clinical indicator, MHS has defined an upper benchmark ("ultimate goal"), which is the level of performance MHS aims to achieve within a 3–5 year period. For every given year, an organizational target is set, based on prior progress towards the upper benchmark and an estimation of resources and capabilities. A unique mechanism helps to adjust a tailor-made unit target, taking into account the unit’s diverse member populations and their socio-economic; personal and social characteristics. Weighted unit's grades (UGs) for all indicators are calculated and contribute to the unit's "overall grade" for the purpose of recognition and modest reward. This was done as follows:

**Step 1. Formulating an indicator factor (IF):** Since each indicator demonstrates a different range of performance, we formulated a factor for each indicator (i), calculated yearly as follows:

$$ \frac{\mathrm{IFi} = \left(\mathrm{M}\mathrm{H}\mathrm{S}\hbox{'}\ \mathrm{annual}\ \mathrm{target}\ \hbox{--}\ \mathrm{end}\ \mathrm{of}\ \mathrm{previous}\ \mathrm{year}\ \mathrm{M}\mathrm{H}\mathrm{S}\ \mathrm{performance}\right)}{\left(\mathrm{M}\mathrm{H}\mathrm{S}\hbox{'}\ \mathrm{ultimate}\ \mathrm{goal} - \mathrm{end}\ \mathrm{of}\ \mathrm{previous}\ \mathrm{year}\ \mathrm{performance}\right)} $$

**Step 2. Calculating the unit target**, using the following formula for each clinical indicator:

UTi = IFi *(MHS' upper benchmark - the unit's last year^†^ performance) + the unit's last year† performance (^†^ December 31).

**Step 3. Calculating a Unit's Grades (UG)**

UGi = (0.3 *% of achieving the upper benchmark) + (0.7 *% of achieving the tailor-made target). These rates (30% and 70%) were assigned based on the idea to encourage excellence beyond the tailored targets.

**For example**: MHS's upper benchmark (for the next 3–5 years) for mammography screening was 80%; MHS' target for the upcoming year is assigned to 70%; MHS' end of last year performance was 60%.

$$ \frac{\mathrm{Mammography}\ \mathrm{IF}=\left(70\hbox{--} 60\right) = 0.5}{\left(80\hbox{--} 60\right)} $$

The unit's last year performance in Mammography screening was 50%

$$ \mathrm{U}\mathrm{T}\ \mathrm{mammography} = 0.5\left(80\hbox{--} 50\right) + 50 = 65\% $$

$$ \mathrm{U}\mathrm{G}\ \mathrm{mammography} = 0.3\ \left(65/80\right) + 0.7\ \left(65/65\right) = 0.94 $$

### Reports and rewards

Performance data at the branch (unit), region and organization levels were distributed monthly to all managers and caregivers. Quarterly and yearly reports analyzed temporal trends and gaps in performance between units serving different sub-populations. These same reports were used to identify the need for process redesign, and to encourage the design of tailor-made local interventions [21].

Rewards were mainly based on organizational acknowledgements to managers who achieved higher scores. Every year, in an organizational managerial conference, these managers were publicly announced. There were no sanctions on those branches who did not meet the desired scores, however, there was a managerial pressure to improve performance.

## Methods

### Analyzing trends in clinical performance and between sub population groups

#### Measurements

In order to evaluate changes in performance over time, six indicators, for which definitions were not changed over time stable and each patient's data was fully recorded, were selected: 1) mammography; 2) colorectal cancer screening; 3) HbA1C performance; 4) diabetes adequate control; and 5) diabetes poor control (Table 1). These indicators were compared for the years 2003–2009 and analyzed for members' socio-economic and ethnic characteristics (Israeli Arabs and Israeli non-Arabs).

### Statistical methods

For each indicator, rates were calculated by dividing the number of eligible patients who utilized the service or achieved the treatment goal by the number of those who met the eligibility criteria for the service.

To assess the significance of the trends over time (2003–2009), we estimated linear regression models for curve fit for each of the selected indicators.

The Curve Estimation procedure generates a time variable in which the period of time between cases is uniform. If Time is selected, the dependent variable should be a time-series measure. Time-series analysis requires a data file structure, in which each case (row) represents a set of observations at different times, and the period of time between cases is uniform [22].

*Dependent variable:* each indicator’s rate.

*Independent Variable*: Socio-Economic Rank (SER) and ethnicity: Israeli Arabs vs. non-Israeli Arabs

*Socio-Economic Rank* (SER) was defined by the rank (1–20 scale) of members' area of residence (based on Israeli Census Bureau data), and then categorized into the lowest (1–5) and highest (16–20) categories [23].

*Ethnicity*: Since Israeli HMOs do not have access to personal ethnicity data, Israeli Arabs were identified by the ethnicity of their residence as recorded in the National Census [23].

## Results

The study population (2009) ranged between 153,785 mammography eligible women and 54,095 adults diagnosed with cardiovascular disease (CVD) who were targeted for decreasing LDL cholesterol (Table 1).

Overall organizational performance demonstrated considerable improvement over the years, which was statistically significant in all indicators except for adequate diabetes control (Table 2). The most considerable improvement over time was demonstrated by colorectal screening (147% from 2004 to 2009) and adequate LDL control in patients with cardio-vascular disease (90% improvement during the study period).

**Table 2:** Performance rates (%) for Selected Clinical Indicators, 2003–2009

Indicator	2003	2004	2005	2006	2007	2008	2009	P for Time Trend
Mammography	51.3	52.7	63.1	67.7	65.3	70.6	73.8	0.001
Colorectal cancer screening	NM^a^	15.6	19.0	27.7	30.4	33.6	38.5	<0.001
HbA1C Performance	NM	89.7	90.2	91.6	91.7	91.3	93.1	<0.001
Diabetes adequate control	53.2	46.7	56.0	54.6	55.9	59.6	59.7	0.035
Diabetes poor control	NM	13.0	11.1	11.7	11.1	9.3	9.2	0.010
Adequate LDL control in patients with cardio-vascular disease	33.0	35.9	45.9	56.3	59.6	61.6	62.8	0.001

^a^NM: Not measured

Performance of lowest SER members improved to a larger extent in comparison to members from highest SERs in all measures except for blood examination of HbA1C levels. As a group, members of Arab ethnicity achieved a larger relative improvement in comparison to non-Arab members (Table 3).

**Table 3:** Percent change, 2003 to 2009, by SER and ethnicity

Indicator	SER 1–5	SER 16–20
Mammography	40.0	29.0
Colorectal cancer screening	77.0	55.0
HbA1C Performance	−35.0	10.0
Diabetes adequate control	13.0	10.0
Diabetes poor control	−40.0	−51.0
Adequate LDL control in cardio-vascular patients	35.0	31.0
Indicator	Arabs	Non Arabs
Mammography	56.0	41.0
Colorectal cancer screening	89.0	65.0
HbA1C Performance	11.0	9.0
Diabetes adequate control	20.0	12.0
Diabetes poor control	−37.0	−57.0
Adequate LDL control in cardio-vascular patients	37.0	33.0

## Discussion

The first objective of this article is to describe a unique module built-in the performance measurement system, for setting tailor-made targets per unit. The allocation of these targets intended to adjust for the current level of performance, which in turn reflected the case mix of the units, as well as their personal and social characteristics to improve care. By expecting units with lower levels of performance to achieve a larger absolute (and relative) improvement, the message to regional managers was a demand to allocate more resources to the weaker branches, in order to gradually close the gap. This in turn was transmitted to front-line caregivers. A request to provide special attention to less achieving units pushed the formulation of local solutions to overcome barriers that potentially prevented weaker populations from achieving optimal health outcomes. An example of such a productive local activity, supported by organizational infrastructures, is the case of increasing breast cancer screening among Arab women [21]. The managerial demand for preferential allocation of resources to the weak units was effective in reducing some of the disparities in health measures. In fact, the module of tailored targets resulted in "lifting the floor rather than pushing up the ceiling". Putting more emphasis on the relative improvement of weaker units was also reflected, as allocated in the scoring formula, to a greater extent, to the units for achieving their tailor-made target - i.e., for making a greater relative improvement (0.7 of the score for each indicator). However, in order to praise excellence as well, high absolute performance was also rated, although to a lesser extent (0.3 of the score).

The second objective of this article was to present analysis of the effect of the above described concept, on care quality and equity. From the year 2003 to 2009, most of the selected quality indicators demonstrated a significant and steady improvement. Of special value is the finding that members who lived in low socioeconomic neighborhoods or in Arab settlements/local authorities achieved a relatively larger improvement, in comparison with members of higher socioeconomic groups or non-Arabs.

Over the years, MHS has paid attention to and invested resources in care quality improvement. This was done by sending a clear managerial message, distributed by the target-based measurement system. Transparency of performance, frequent internal reporting and competition over recognition and modest rewards (additional budgets for social events for the unit's staff), all contributed to a clear unequivocal organizational "voice".

This message was facilitated by MHS' Quality Infrastructure, consisting of a central quality body, as well as multidisciplinary "quality teams" at the regional and branch levels. Teams were provided with training, tools and a framework for organizational learning from across units while implementing quality improvement initiatives such as breast cancer prevention program among Arab women [21].

It should be noted that tailoring of targets is only one, and definitely not the sole mechanism, for improving equity of health care and health outcomes. Experiencing success in some reduction of disparities indeed created organizational discourse on equity and equality issues, and raised top managerial concern and belief that this might be feasible. From a long-term perspective, implementation of the measurement system has made "quality" integral to the MHS organizational discourse. This dialogue, supported by a set of validated data, has contributed a new "language". Terms like quality gap, data analysis, prioritization of domains for intervention, effective methods for improvement and reduction of health disparities have all helped create a new organizational culture within MHS. Medical directors, as well as nonmedical administrators, all perceived quality improvement as a crucial element in their role definitions. Quality issues have consequently captured an increasing portion of the managerial dialogue conducted with primary care physicians and other health professionals.

Performance measuring systems are used in many health organizations around the world. To illustrate, the American Veterans Affairs (VA) healthcare system, has undergone various transformations in an attempt to improve quality of care, including implementation of performance measurement, focusing on high-priority conditions such as diabetes and coronary artery disease. In earlier published studies, the VA reported a steady and significant increase in all the selected measures [24–27], including higher performance levels, when compared with non-VA settings [4, 27]. However, the VA reported that despite the improvement in quality of care, racial disparity persists for important Clinical Outcomes [27]. Our statistical analysis reveal that, disparities between sub population groups, in the presented clinical measurements, were reduced. It is for sure, that the way we weighed unit's performance and encouraged them to take bold steps and invest in intervention program, to close gaps, has to do with this reduction, although no statistical model could estimate for this unique formula as an independent variable.

Our positive statistical findings should take several limitations into consideration:

First: the lack of a control group. The PMS was initiated as an organizational system which covers all units. As a result, there are no units that can serve as a control group. What we can see, are the differences between units and subpopulation groups, in performance and intermediate outcomes, for which the tailored targets try to compensate and adjust.

Second, due to technical barriers such as the insufficient timely flow of hospital data on health outcomes, we were limited to the use of process and intermediate outcomes to evaluate the quality of care provided to MHS members. The use of long-term outcome measures, such as rate of amputations among diabetics or disease-related mortality, was precluded.

Third, most quality indicators are sensitive to patient characteristics for their validity [28]. Israeli HMOs face legal constraints on the collection of selected types of personal socio-demographic data, such as income or ethnicity. We therefore used proxies, such as the rank of the geographic statistical index of the members' area of residence. And third, a design of case control study would have provide a better understanding the association between the PMS and the health outcomes observed here. However, the Israeli healthcare system, which is universal insist, by law, on equity and equality. Thus, there is no way to provide unequal services just for the sake of the research.

Fourth: we analyzed six indicators, for which the definitions has not been changed over time and the patient's information was fully recorded. These indicators do not necessary represent the other indicators.

## Conclusion

The Performance Measurement System described in this article, with its unique built in formulas, adjusting for the relative level of performance, has a potential of serving as a locomotive, pulling the quality system toward its goals. The concept of management by assigning tailor-made targets to healthcare services units and rewards for achieving those targets, proved effective in improving care, while reducing disparities.

## Abbreviations

CEO: Chief executive officer
CMO: Chief medical office
CVD: Cardio-vascular disease
HEDIS: Healthcare effectiveness data and information set
HMO: Health maintenance organizations
IF: Indicator factor
MHS: Maccabi healthcare services
PMS: Performance measurement system
SERs: Socio-economic ranks
UGs: Weighted unit's grades
VA: The veteran affairs
